# Challenging regulations: Managing risks in crop biotechnology

**DOI:** 10.1002/fes3.60

**Published:** 2015-06-22

**Authors:** Huw D. Jones

Alongside other aspects of agriculture, plant breeding is pivotal to securing crop yields necessary to meet the growing demands for human food and animal feed. In addition to the important targets of yield, nutritional quality and resilience to abiotic stresses, breeding for resistance to pests and diseases will become even more critical as the availability of plant protection products is further diminished by regulation and/or the lack of new active ingredients coming to market (Chapman 2014) and by consumer preferences for fewer inputs. In this regard, it is fortuitous and timely that future crop genetic improvement will be aided by several developments in plant breeding that are underpinned by the massive increase in DNA sequence information flowing into databases from new methods of reading DNA that were developed in the mid 2000's (Moorthie et al. 2011). A stark example of this revolution in sequencing speed is seen in the progress of rice genomics where, after about three years work, the first draft genomic sequence of rice was published in 2002 (Goff et al. 2002; Yu et al. 2002). Yet, little over ten years later, IRRI published the genomic sequence of 3000 different rice varieties (Alexandrov 2015). This step‐change in sequencing and bioinformatics resulted in the generation of massive data sets that can be mined to give information about the genetic location and function of specific alleles which in turn can be used to inform and facilitate crop improvement via conventional breeding methods or via a spectrum of rapidly evolving molecular breeding technologies and concepts of synthetic biology. Two such technologies close to commercialization are genome editing and interorganism silencing which are discussed below. However, the plant breeder faces significant challenges to fully integrate these novel approaches to produce new varieties because of ambiguities and uncertainties surrounding risk assessment and regulation.

## Advanced Breeding Techniques

Methods used to breed better crops have never stood still but over the last 50 years there has been a major shift toward using advanced, laboratory‐based techniques in the production of new crop varieties. These techniques fall broadly into two categories, those that aid the rapid selection of desirable individuals in a breeding programme and those used to increase the genetic variation available to breeders. Advances in the first category, such as seed‐chipping devices and rapid DNA fingerprinting methods that allow the high throughput use of new genetic markers to screen large breeding populations for successful introgression of specific allelic combinations, do not on their own, pose any new hazards and are rightly not formally included in any regulatory framework. However, biotechnology regulations in many territories do capture some of the latter category of breeding activities but there is a lack of logic, consistency and clarity in their implementation around the world. For some newer technologies that were not envisioned when the regulations were created, there is significant uncertainty over what is regulated and what is not. This lack of guidance by risk managers stifles new technologies before their potential can be explored. Below I outline some of the challenges facing breeders and regulators in the rapidly evolving field of biotech crops.

## Genome Editing

Over that last few decades, the discovery and further refinement of a range of targeted endonucleases has given rise to the truly disruptive technology of genome editing which is the ability to cut and edit by insertion, substitution, or deletion, predetermined sequences in genomes. The enzymes and recognition domains that carry out this site‐specific DNA cleavage are found naturally in eukaryote and prokaryote microorganisms. The main programmable nucleases that are used for research today are: Zinc‐finger nucleases (ZFN), Transcription activator‐like effector nucleases (TALENs), and Clustered regularly interspaced short palindromic repeats (CRISPRs). A variant on these methods is Oligonucleotide‐directed mutagenesis (ODM) which does not incorporate a nuclease to cleave DNA but instead uses short, chemically synthesized DNA/RNA oligonucleotides possessing similarity to the target sequence except for the few bases to be edited that are incorporated into the genome during repair. Meganucleases (MNs) are naturally occurring restriction enzymes which also possess highly specific target sites, but are not easily targeted to different DNA sequences and for this reason are losing ground to the programmable, two‐component systems. Site‐directed nucleases (SDN) generate mutations by cutting the targeted DNA and allowing the cell's repair machinery to rejoin the ends. In this case, where there is no overlap to guide the repair, the cell uses a mechanism known as nonhomologous end‐joining (NHEJ) which is error‐prone and generates mutations. This is significant from a regulatory point of view because the resulting mutations are indistinguishable from the random mutations generated by the plant due to normal DNA damage and from the potentially more disruptive sequence changes generated by intentional to exposure of seeds to radiation or chemical mutagens as part of mutation breeding programmes, which are excluded from most GMO regulations (Jones 2015).

Despite the regulatory uncertainty, genome editing holds considerable promise for crop improvement. Using basic NHEJ‐mediated target mutation, the initial trait goals would be restricted to gene knockouts or very simple edits. Some obvious crop targets are the removal or biochemical alteration of toxins, allergens, acrylamide, or antinutrients in grain; the blocking of pest or pathogen recognition signals to give resistance to pests and diseases; or the manipulation of functional and end‐use qualities such as soluble and insoluble fiber. However, it is also feasible to drive larger and more predictable changes to a predetermined genomic site by simultaneously supplying excess copies of short “repair template” DNA that possesses an overlap with the DNA flanking the cut site along with the SDN. In this case, the cell can utilize homologous recombination to repair the break by incorporating the repair template which can be engineered to make highly specific edits to the original sequence. This concept can also be used to repeatedly direct whole transgenes to a predetermined safe‐harbor location in the host genome thus removing the insertion site variable from conventional transformation (Ainley et al. 2013). Recently a Chinese laboratory produced broad‐spectrum mildew‐resistance in wheat by knocking out MLO, a genetic locus suspected of suppressing plant defence against mildew. Plants where all six alleles of MLO were successfully knocked out by TALENs or CRISPR were highly resistant to mildew (Wang et al. 2014). The first commercial application of genome editing was developed by Cibus Global, a San Diego‐based company who describe themselves as a precision gene editing firm. In March 2014 they received regulatory approval from Canadian Food Inspection Agency and Health Canada to commercialize a novel Sulfonylurea tolerant Canola generated using their proprietary Genome Repair Oligonucleotide technology (Canadian Food Inspection Agency 2013, AgCanada News 2014). It is expected to be launched for cultivation in Canada in 2016 (Cibus Press Release 2014).

## RNAi‐Mediated Silencing

Even before the molecular mechanisms of RNA interference were discovered, plant breeders were unwittingly selecting for traits in crops mediated by RNA‐based mechanisms. For instance, the light yellow color of modern soy varieties is due to a natural‐occurring change that silences chalcone synthase expression and blocks production of anthocyanin pigments that give wild soy seeds their normal brown or black appearance (Todd and Vodkin 1996). Techniques to silence the expression of specific genes are well‐established and there are many examples of plant varieties that are fully commercialized or close to market. For example, two soy varieties (Monsanto's Vistive Gold and DuPont‐Pioneer's Plenish^™^) possess high oleic/low linolenic oil giving better heat stability for frying, longer fry life and improved flavor of fried products produced partly through a gene silencing effect. Arctic Apples (Okanagan Specialty Fruits) which received regulatory approval from APHIS in March 2015 (USFDA 2014) have less of the enzymes that cause browning when apples are cut. Apple browning is caused when phenols are produced through the action of the enzyme polyphenol oxidase which has been reduced to only 10% of its normal levels in these GM apples using RNAi silencing. In another significant development the J.R. Simplot Company received a determination of nonregulated status from USDA in Nov 2014 (USDA 2014) and recently publicized its commercial rollout plan for the Innate^™^ potato (Simplot 2014) that is engineered for low‐acrylamide potential and reduced black spot bruising. Commercialization will be managed within a tightly controlled network of growers and processors but Simplot declare they have no plans to begin any sales in the US before 2015. Innate^™^ potatoes use DNA sequences that originate only from potato to silence the genes; asparagine synthetase‐1, polyphenol oxidase‐5, potato phosphorylase L, and the starch‐associated R1 gene resulting in potatoes with reduced free asparagine, a lower content of reducing sugars and with a nonbrowning phenotype resulting in tubers with reduced black spot bruising. The concept of using only genes from crossable species in genetic manipulations is called Cisgenesis (Holme et al. 2013) and can result in GM crops that require less data for risk assessment (EFSA GMO Panel 2012).

These “within‐plant” (intra‐organism RNAi) silencing effects are already seen in many commercial biotechnology crops but on the horizon are a new family of crops that use a novel silencing approach that could challenge the GMO regulations. These plants still generate a silencing signal but rather that targeting a gene within the plant itself, the silencing effect is aimed at a gene in an attacking pest or pathogen (inter‐organism RNAi). If successful, and if the potential risks associated with “off‐target” gene silencing can be addressed, this cross‐species or host‐induced gene silencing could largely replace chemical insecticides and fungicides in the control of major crop pests.

## Other New Breeding Techniques

Grafting non‐GM fruit‐bearing scions onto GM root‐stocks that produce, for instance, an antimicrobial peptide could protect fruit vulnerable to specific diseases. To test this hypothesis, transgenic Thompson Seedless grape expressing the Shiva‐1 lytic peptide gene was treated as a rootstock. Nontransgenic Cabernet Sauvignon and Thompson Seedless grape scions were grafted onto this rootstock (Dutt et al. 2007). The presence of Shiva‐1 peptide in xylem sap of the scion was confirmed by ELISA and demonstrates the potential to protect fruit from Pierce's disease which is caused by the xylem‐limited bacteria, *Xylella fastidiosa* (Dutt et al. 2007). The edible parts of the plant are non‐GM but they are exposed to a recombinant protein produced by GM roots. Under current EU GMO regulations for cultivation, the GM/non‐GM chimeric plant would be considered a GMO and risk assessed. However, it is less certain whether or how the import into the EU of such non‐GM fruit would be regulated.

## Other New Breeding Techniques that also have an Uncertain Regulatory Status in the EU include:

1. RNA‐dependent DNA methylation (RdDM) which incorporates epigenetic changes in the genome but leaves no recombinant DNA is in the final product.
2. Reverse breeding that involves an intermediate step where recombinant DNA is present in the plant to suppress meiosis but where no foreign genetic material remains in the end product.
3. Crops with altered traits due to transient (temporary and nonintegrated) expression of recombinant DNA.

## Regulatory Frameworks for GM Plants

Several international bodies (including OECD, FAO, WHO) have produced fundamental principles for the food/feed and environmental risk assessment of crops made using modern biotechnology. In 2003 the Codex Alimentarius Commission of the FAO/WHO adopted a set of “Principles and Guidelines on foods derived from biotechnology” to help countries coordinate and standardize regulation of GM food to help ensure public safety and facilitate international trade. This said, the regulatory frameworks that govern breeding and biotechnology have evolved differently in the various regions of the world and this has already created anomalies in the treatment of new varieties. It seems likely that this will get even more difficult with international food/feed supply chains and as new plant breeding techniques are used commercially. Especially challenging for free trade will be the scenario where a product of a new technology will be deemed a conventional crop in one country, with no requirement for risk management, and a GMO, with full regulatory oversight, in another.

Canada has taken a “novel traits” approach and regulates new varieties on the basis of the risks posed by its characteristics regardless of the breeding methods used. These are plants that contain a trait which is both new to the Canadian environment and has the potential to affect the specific use and safety of the plant with respect to the environment and human health. These traits can be introduced using biotechnology, mutagenesis, or conventional breeding techniques (Canadian Food Inspection Agency 2015).

The USA developed the “Coordinated Framework for Regulation of Biotechnology” (Office of Science and Technology Policy 1986) which was a regulatory policy framework to adapt their existing food safety and crop protection laws to also cover biotechnology. As a result, no single statute or federal agency governs the regulation of biotechnology products. The regulation of GM crops is divided between APHIS (the Animal Plant Health Inspection Service of the USDA), EPA (the Environmental Protection Agency) and the FDA (Food and Drug Administration) each with its specific areas of responsibility. One of the main tenets of the policy was to focus on the product of genetic modification (GM) techniques, not the process itself. However, in at least one respect, APHIS does use process rather than product as a regulatory trigger when they decided that Scott's GE Kentucky bluegrass variety does not fall under their authority for regulation even though it is genetically‐engineered to be resistant to the herbicide glyphosate. This decision is based on the process of transformation; Scott's bluegrass was transformed using biolistics and not using the “plant pest” Agrobacterium but also on the view that a single gene insertion does not create a new species of Kentucky bluegrass that is itself a plant pest or noxious weed (Montgomery 2013).

**Figure 1:**
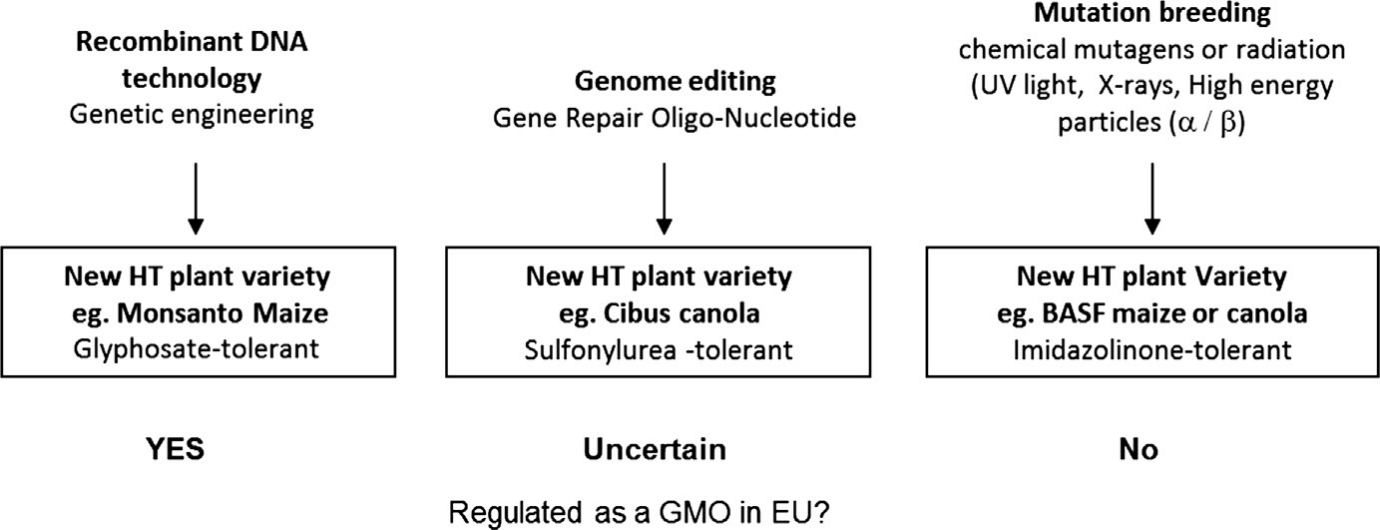
Three processes, with examples that have generated commercial herbicide tolerant crops and the EU GMO regulatory implications.

In the EU crops bred using biotechnology are covered by the Deliberate Release Directive (2001/18/EC) which governs the release of GMOs to the environment including research trials (European Parliament 2001). This directive is based wholly on the processes used to alter the plant's genome and covers any organism (except humans) that has been altered in a way that does not occur by natural mating or genetic recombination, implying that it is the process, rather that the products, that pose the risk. However, as we learn more about the natural plasticity of plant genomes and the behavior of transgene insertion on host genomes, a better trigger for risk assessment would be the trait not the process that generates it. Interestingly, EC 2001/18 explicitly excludes from the regulations, varieties resulting from mutation breeding or certain examples of cell or protoplast fusion. Although these techniques do not incorporate the use of recombinant DNA, they can still generate major and unpredictable changes to plant genomes which is one of the potential hazards that the EC requires to be identified, characterized and risk‐assessed in GM plants. Ironically, although these excluded techniques are not regulated, they can be used to produce the same altered traits as conventional genetic modification. This exposes a fundamental illogicality in the regulations which could be further compounded if the EC decides to regulate plants derived from genome editing as GMOs rather than products of mutation breeding. As an example, take herbicide tolerance (HT), the most commonly GM crop trait and one implicated in major changes in agricultural practice and for accelerating herbicide resistance in weeds. There are now at least three different methods to generate a HT crop; conventional genetic modification, conventional mutation breeding, and genome editing which has elements of both methods. Current EU regulations mean the three products would be regulated differently from each other (Fig. 1). As discussed above, it is also likely that they would also be treated differently depending on where they were cultivated.

For agriculture to fully exploit the revolution in plant genomics, bioinformatics, and molecular genetics, plant breeders as well as consumers and other stakeholders in the various agricultural, food, and feed industries, need transparent and logical regulations that take a proportionate account of risks and benefits. The international trade in bulk commodity, as well as speciality crops dictates that these regulations need to be harmonized and that approvals for new varieties are synchronized.

## Conflict of Interest

None declared.
